# Biotechnologies for bulk production of microalgal biomass: from mass cultivation to dried biomass acquisition

**DOI:** 10.1186/s13068-023-02382-4

**Published:** 2023-08-29

**Authors:** Song Qin, Kang Wang, Fengzheng Gao, Baosheng Ge, Hongli Cui, Wenjun Li

**Affiliations:** 1 Yantai Institute of Coastal Zone Research, Chinese Academy of Sciences, No. 19, Chunhui Road, Laishan District, Yantai, 264003 Shandong China; 2 University of Chinese Academy of Sciences, Beijing, 100049 China; 3 Bioprocess Engineering, AlgaePARC, Wageningen University, P.O. Box 16, 6700 AA Wageningen, Netherlands; 4 College of Chemical Engineering and Center for Bioengineering and Biotechnology, China University of Petroleum (East China), Qingdao, 266580 China; 5 Laboratory of Sustainable Food Processing, ETH Zürich, 8092 Zurich, Switzerland; 6 Laboratory of Nutrition and Metabolic Epigenetics, ETH Zürich, 8603 Schwerzenbach, Switzerland

**Keywords:** Microalgal biotechnology, Biomass, Cultivation, Harvesting, Drying, Biological contaminant control

## Abstract

Microalgal biomass represents a sustainable bioresource for various applications, such as food, nutraceuticals, pharmaceuticals, feed, and other bio-based products. For decades, its mass production has attracted widespread attention and interest. The process of microalgal biomass production involves several techniques, mainly cultivation, harvesting, drying, and pollution control. These techniques are often designed and optimized to meet optimal growth conditions for microalgae and to produce high-quality biomass at acceptable cost. Importantly, mass production techniques are important for producing a commercial product in sufficient amounts. However, it should not be overlooked that microalgal biotechnology still faces challenges, in particular the high cost of production, the lack of knowledge about biological contaminants and the challenge of loss of active ingredients during biomass production. These issues involve the research and development of low-cost, standardized, industrial-scale production equipment and the optimization of production processes, as well as the urgent need to increase the research on biological contaminants and microalgal active ingredients. This review systematically examines the global development of microalgal biotechnology for biomass production, with emphasis on the techniques of cultivation, harvesting, drying and control of biological contaminants, and discusses the challenges and strategies to further improve quality and reduce costs. Moreover, the current status of biomass production of some biotechnologically important species has been summarized, and the importance of improving microalgae-related standards for their commercial applications is noted.

## Introduction

In applied biology, the term ‘microalgae’ usually refers to prokaryotic cyanobacteria and eukaryotic microalgae [1]. These organisms are widespread and can be found in almost all ecosystems, from extremely cold polar regions to dry deserts [2]. Photosynthetic microalgae provided the Earth with the initial oxygen supply, creating an environment conducive for the evolution of various forms of aerobic life over time. Furthermore, microalgae are important CO_2_ consumers and major producers because of which they have attracted attention in recent decades as one of the most effective converters of solar energy into biomass.

### From natural resources to artificial culture

**Fig. 1:**
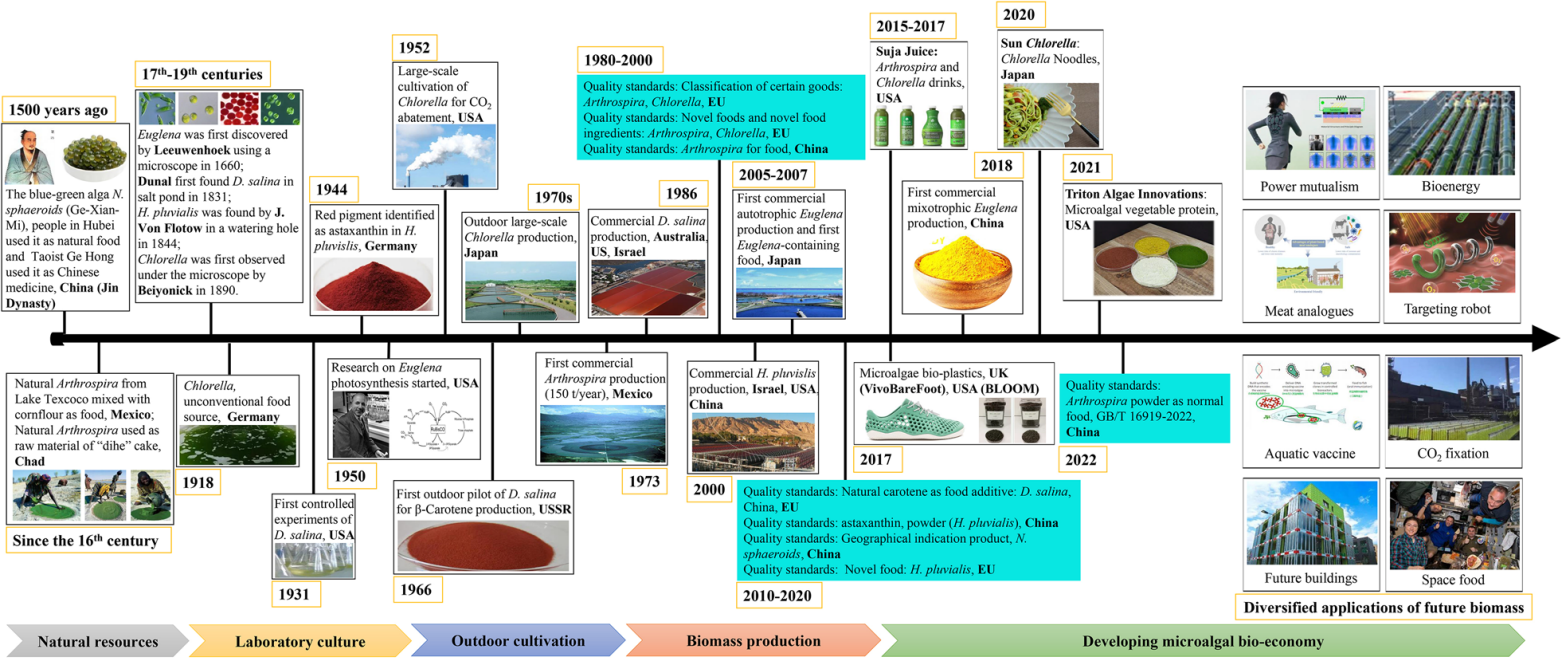
Milestones in microalgal biotechnology and large-scale cultivation: from natural resources to industrial biomass production and diverse applications

Microalgal biomass has been used since antiquity (Fig. 1). Initially, it was used to cope with food shortage. For centuries, natural biomass of the blue–green alga, *Arthrospira*, was harvested in certain environments of alkaline soda lakes in countries, such as Chad or Mexico, and was used as a food supplement [2]. During the rule of the Jin Dynasty in China (about 1500 years ago), another cyanobacterium, *Nostoc sphaeroides* (known as Ge-Xian-Mi), was collected for food or traditional Chinese medicine [3]. However, the ancients were unable to cultivate *N. sphaeroides* and *Arthrospira* widely as traditional food crops due to insufficient production technology. Early microalgae work on artificial culture started in Europe in the mid-nineteenth century. A small-scale laboratory culture was started by a German scientist [4]. Over the next few decades, microalgae began to be used as experimental material for basic studies on plant physiology and ecosystem due to their natural advantages, such as high growth rates, high light and efficiency and ease of cultivation in the laboratory [4, 5].

### From lab culture to outdoor cultivation

In the early twentieth century, algae researchers considered the large-scale cultivation of microalgae as a food substitute in response to the food crisis [4, 6, 7]. However, although cultivation of pure lines of microalgae such as *Chlorella* in the laboratory has been skillfully mastered, the bottleneck in outdoor cultivation lies in the lack of facilities and technologies of for large-scale production. The first attempt to translate the biological requirements of photoautotrophic biomass culture into engineering specifications for large-scale cultivation was achieved during 1948–1950 by workers at the Stanford Research Institute, San Francisco, USA [8]. Notably, this problem was almost simultaneously tackled in Germany [9]. In 1951, the construction and operation of a *Chlorella* pilot plant for the Carnegie Institution (Washington, USA) was undertaken by Arthur D. Little, Inc. [8]. The *Chlorella* pilot plant showed that large-scale cultivation of microalgae was technically feasible, although the expenses had to be reduced [8]. Subsequently, Japan began to study the large-scale cultivation technologies of *Chlorella* for developing functional food material in the 1960s [10]. Some progresses have also been made in industrial-scale processes for heterotrophic cultivation of microalgae. For example, in the late 1970s, *Chlorella* producers in Japan and Taiwan attempted to supplement acetate or glucose as carbon and energy sources to heterotrophically cultivate *Chlorella* in stainless steel tanks [11, 12]. Notably, in the 1970s, another microalga, *Arthrospira*, was used in large-scale outdoor cultivation near the alkaline soda lakes in Mexico, and production reached 1000 kg day^−1^ in 1974 [13]. In the 1980s, large-scale cultivation of *Dunaliella* was established in the USA and Australia to produce β-carotene [14]. By the end of the twentieth century, thanks to the progress in large-scale cultivation processes, several microalgal species were in commercial production or were at the pilot stage, and microalgae cultivation became popular worldwide.

### Biotechnological contributions to biomass production

In the twenty-first century, the global demand for microalgae is dominated by food, health products and feed [15–18]. However, these demands require further increase in biomass production and strict control of product quality, as well as the development of production strains. Various techniques have been investigated to achieve the above objectives. For example, the ultrahigh‐cell‐density heterotrophic cultivation technique was developed for two *Chlorella* species and *Scenedesmus acuminatus*, which provided an important technical foundation for promoting its industrial application as an alternative high-quality protein source of food and feed [19–21]. In recent years, China has been able to produce nearly 10,000 metric tons of *Arthrospira* per year through improved cultivation techniques and screening for low-temperature tolerant algae strains [22]. Some species with stricter growth conditions have been successfully produced on an industrial scale in a cleaner way due to the innovation of photobioreactor and cultivation techniques. For example, 25,000 L outdoor photobioreactors have been set up for commercial production of astaxanthin from *Haematococcus pluvialis* in 2000 [23]. In Japan, Euglena Co., Ltd. (Tokyo, Japan) has successfully completed the world’s first large-scale cultivation of *Euglena gracilis* for the production of health foods; its commercial cultivation began in 2007 after the improvement of the harvesting and drying techniques suitable for *Euglena* cells [24]. More recently, a high cell-density process of sequential heterotrophy–dilution–photoinduction (SHDP) for producing *Euglena* and *Chlorella* biomass has been patented in China. In addition to cultivation techniques, other processes, including harvesting, drying, and control of biological contaminants also guarantee the production of microalgal biomass on an industrial scale at acceptable cost. Owing to the continuous contribution of biotechnology, the global output of microalgal biomass is now close to 20,000 metric tons [6, 25–30].

### Challenging microalgal industry and technology

The commercial production of microalgae driven by biotechnology is considered a new agricultural model, which can provide sustainable raw materials for hundreds of emerging products and become the driving force of global economic growth. However, the fact is that the various processes of microalgal biomass production have a number of economic or technical drawbacks. In particular, high production costs remain an important factor limiting biomass production. In addition, there is a lack of understanding of biological contaminants in the cultivation process and the challenge of active ingredient loss during biomass drying has not been fully addressed. Standards related to microalgae and its products are important to ensure safety and quality. Regrettably, the microalgae industry still lacks sound standards. These issues involve the research and development of low-cost, standardized, industrial-scale microalgal production equipment and the optimization of production processes, as well as the urgent need to increase the research on biological contaminants and microalgal active ingredients. Briefly, the future of microalgal biotechnology is still challenging.

**Fig. 2:**
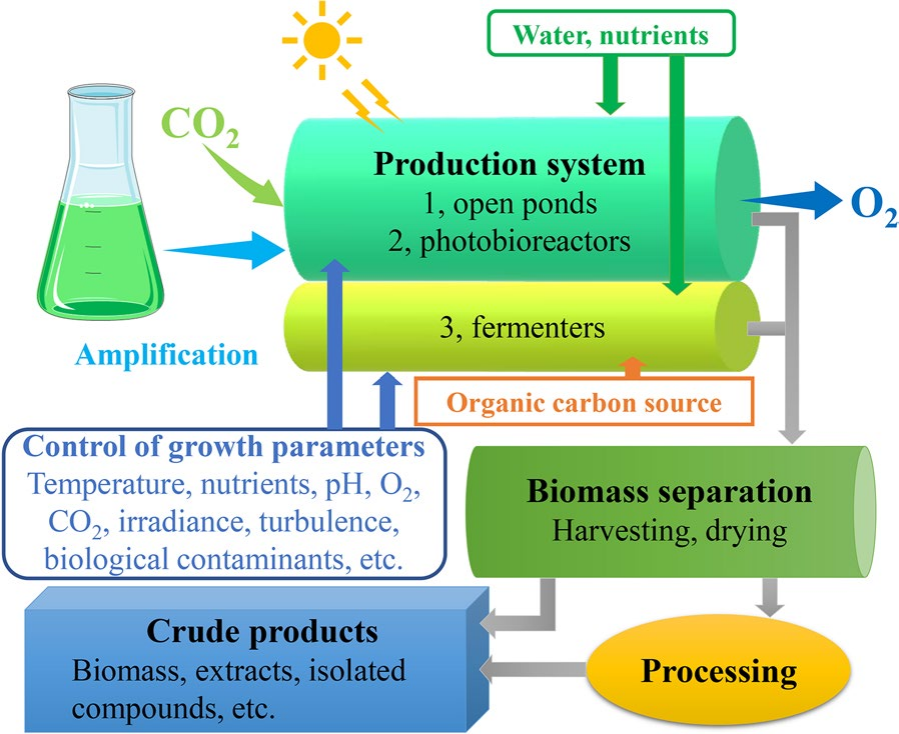
Schematic diagram of microalgal biomass production. Microalgae are grown in a cultivation unit in an aqueous mineral medium under illumination, and nutrient and carbon source (CO_2_, acetate or glucose) supply; subsequently, the biomass is separated from the medium (harvesting) and drying for further application

Currently, there are a number of excellent reviews on microalgal cultivation and biomass utilization strategies [30–32]. However, a comprehensive assessment of the whole process of biomass production is lacking. The novelty of this paper lies in the systematic presentation of key technologies for the production of microalgal biomass, from algal cultivation to dried biomass production. A schematic diagram showing microalgal biomass production is summarized in Fig. 2. Furthermore, some cost-effective technological strategies for biomass production are discussed, and the importance of improving microalgae-related standards for the further development of microalgal industrialization is highlighted.

## Biomass production systems: from phototrophic to high cell-density heterotrophic cultivation

### Widespread photosynthetic mass cultivation-open pond systems

#### Traditional open pond systems for microalgal cultivation

The original idea of photosynthetic mass cultivation of microalgae was developed in Germany in the early 1940s to produce lipids from diatoms [11]. However, this plan was aborted because of the Second World War. Systematic research into photoautotrophic mass cultivation of microalgae began in the late 1940s at the Stanford Research Institute and Carnegie Institution for developing novel food [8]. Currently, the photoautotrophic growth mode is still the most commonly used technique used in microalgal industries, which contributes to the vast majority of global biomass. The advantages and disadvantages of different microalgal cultivation systems are summarized in Table 1.

**Table 1:** Comprehensive comparison of common microalgal biomass production systems

Cultivation units	Commercial species	Advantages	Disadvantages
Open ponds			
Natural/artificial ponds	*Arthrospira* sp.; *D. salina*	• Low construction and operation costs • Easy to maintain and clean • Large capacity • Mature cultivation technology	• Low light utilization • Sensitive to biological contaminants • Suitable for few species • Evaporation losses and CO_2_ losses • High harvesting costs • Climatic dependence • Larger area requirements
Raceway ponds	*Arthrospira* sp.; *Chlorella* sp.; *D. salina*; *H. pluvialis*; *E. gracilis*		
Circular ponds	*Chlorella* sp.;* E. gracilis*		
Circulation cascades	–		
Enclosed photobioreactors (PBRs)			
Tubular PBRs	*Arthrospira* sp.; *Chlorella* sp.; *D. salina*;* H. pluvialis*	• Larger surface-to-volume ratio • Low CO_2_ losses • Reduced risk of contamination • Smaller area requirements • Prevention of evaporation • Higher cell productivities • High species applicability	• Higher construction and operation costs • Overheating and fouling • Difficult to maintain and clean • High concentration O_2_ accumulation • Cell damage by shear stress • Difficulty in scaling up
Vertical-column PBRs	–		
Flat panel PBRs	–		
Fermenters			
Fermenters	*Chlorella* sp.; *E. gracilis*;* H. pluvialis*	• High growth rate and high productivity • Low or none requirement for light • Cost effectiveness	• Suitable for few species • High organic carbon costs • Sensitive to bacterial contamination • Reduction of intracellular photosynthetically derived compounds

Open ponds are the oldest microalgal cultivation systems. Typically, natural or artificial ponds, raceways, and circular ponds represent open pond systems for microalgae, where algae are cultivated under conditions identical to the external environment [2, 11]. Natural or artificial ponds represent extensive open systems and usually comprise a large pond without special modifications, i.e., stirring and CO_2_ addition. This system has minimal construction and operation costs, although maintaining of monocultures and controlling of environmental parameters is difficult. In addition, lower cell density means lower productivity and increase in the cost of harvesting. For example, artificial shallow ponds (2000–5000 m^2^) used for *Dunaliella* cultivation in Western Australia can only produce 1 g dry weight m^−2^ day^−1^ [2]. Intensive open pond systems on a commercial scale mainly contain raceways and circular ponds. An intensive open pond is smaller than natural or artificial ponds. In this system, some facilities for improving growth conditions of microalgae, such as stirring and CO_2_ supplement devices, are installed [11]. Therefore, sufficient CO_2_ supplement can be provided for microalgal photosynthesis, and stirring enhances the light utilization efficiency of cells. The world's earliest specially modified raceway pond was designed by workers in Germany in the 1950s for evaluating the possibility of biological utilization of CO_2_ from waste gases [9]. This open-air plant consisted of four culture trenches with a fall of 6 mm m^−1^, each 9 m long and 70 cm wide [9]. These trenches were rammed down in loam and were lined with plastic. In addition, devices including pump, centrifuge, collecting vessel, and gas pipeline were installed to control the growth parameters and harvesting. Today’s commercially available raceways largely follow or are improvements upon this design. The circular pond, the most common open system for mass production, was first developed and used in Japan in the 1960s. This system mainly included a rotating arm for mixing and a circular pond with a maximum diameter of 50 m. The design of the open circular pond limits the size to about 10,000 m^2^, because relatively even mixing by the rotating arm is no longer possible in larger ponds. Notably, most of the culture ponds for *Chlorella* cultivation used in Japan are circular in shape and up to 50 m in diameter. However, the cultures in these intensive open systems are usually grown at biomass concentrations in the range of 0.5 to 1 g L^−1^, and the light utilization efficiency of cells is the main limiting factor, which depended on the culture depth and stirring.

#### Circulation cascades

Circulation cascades (i.e., inclined-surface systems) were considered as high cell-density open culture systems for microalgae. The first experimental circulation cascade was designed by Dr. Ivan Šetlík and was built at the Botanical Garden of the Slovak Academy of Sciences in the late 1950s [33]. The system was constructed as stepped arrangement shallow troughs made of reinforced polyester resin. Circulation cascades have several advantages; in particular, microalgal cultures can flow over sloping planes arranged in inclined surface, which allows the culture depth to be controlled at a low level (usually below 1 cm), while the turbulence generated by the device also prevents self-shading of cells. Therefore, high productivities can be achieved easily in this open system [34]. Recent studies using this system for culturing *Chlorella* sp. MUR 268 and *Scenedesmus obliquus* have achieved productivity in excess of 20 g dry weight m^−2^ day^−1^ [35, 36]. However, although circulation cascades are transportable with long working life, higher construction costs limit complete scale up of this system.

#### Challenges of open pond systems

A major challenge of open ponds is the sensitivity to pollutants. Thus, only few species can be cultivated in these ponds for biomass production on a commercial scale, such as *Arthrospira*, *Chlorella*, and *Dunaliella*. The main characteristic of these species is that they can only grow in specific environments, which is hostile to most other competitors. For example, *D. salina* grows in salty water (NaCl concentrations > 20% w/v) and *A. platensis* grows in highly alkaline environments (pH > 9.2). Other microalgae that can be grown in open ponds are rapidly growing dominant species, such as *Chlorella* and *Scenedesmus*. Some other contaminants, including heavy metals and microplastics, are also unacceptable for microalgae cultivation for food and food supplement purpose. Furthermore, massive water loss due to evaporation and low cell concentration and biomass productivity are also the intrinsic disadvantages of open ponds. Therefore, the future techniques for open ponds should address these bottlenecks while maintaining lower production costs. Notably, using open ponds for the production of valuable microalgal products is unlikely to be sustainable or economic, thus, attempts have been made to overcome some of their limitations using closed pond or enclosed photobioreactor systems [11].

### Enclosed photosynthetic mass cultivation–photobioreactor systems

#### Typical photobioreactors

Photobioreactors represent sophisticated and flexible systems working either outdoors or indoors, in which a single species is inoculated to keep a clean culture operation. A photobioreactor is usually equipped with lighting, stirring, CO_2_ addition, and cooling facilities, and it can be better optimized according to the biological characteristics of the microalgal species cultivated. Compared to open systems, enclosed photobioreactors have several advantages, mainly including (i) larger surface-to-volume ratio, (ii) low CO_2_ losses, (iii) reduced risk of contamination, (iv) smaller area requirements, (v) ability to prevent evaporation, and (vi) higher cell productivities. So far, various photobioreactors consisting of glass or transparent plastic tubes, and columns or panels, have been designed using either natural or artificial lighting.

The most commonly used photobioreactors are vertical-column and tubular. The former is a relatively simple system, in which stirring is achieved by air or high concentration of CO_2_ bubbling up from the bottom. Vertical-column reactors described by Cook in 1950 were the first real enclosed systems for microalgae culture [37]. In the 1980s, algae workers evaluated two vertical-column reactors and found that maximum productivity of 20–26 g dry weight m^−2^ day^−1^ for *Chlorella* and *Nannochloropsis* could be achieved in a vertical air-lift photobioreactors [38], while 23 g m^−2^ day^−1^ could be obtained in a vertical glass tube for *Monoraphidium* [39]. Despite the very gentle stirring and good light penetration of this reactor, its potential for scale-up appeared difficult. In fact, vertical-column reactors are commonly used in a seed culture in microalgal factories. The reactors used commercially for biomass production are tubular. Since the pioneering work of Tamiya et al. [40], several tubular reactors have been studied and developed. In general, the tubes are made of glass, plastic, or acrylic as the solar receptor and are arranged as a serpentine loop or as manifold rows. Recirculation of the culture suspension and removal of O_2_ are achieved using a pump (mainly using centrifugal or peristaltic pumps) or an air-lift (injecting a stream of compressed air into an upward-pointing tube) [40]. The cell growth temperature is regulated by a heat exchanger, or by spraying water onto the surface of the photobioreactor. In addition, tubular photobioreactors for commercial production are usually of modular design, which allows easy installation in any open space; for example, the culture systems developed at Batelle in 1980s for the production of polysaccharides from *Porphyridium cruentum* [41]. The two‐plane tubular photobioreactor is another type of tubular reactor first developed by Torzillo et al. [42] in Florence (Italy) for outdoor culture of *Arthrospira*, which led to a high biomass productivity of 30 g dry weight m^−2^ day^−1^. In particular, the largest known microalgae plant using tubular reactors has been established in Pataias (Portugal), with a total-volume of 1300 m^3^ and occupying one hectare of land, operated by A4F-AlgaFuel at the Secil Cement Company, for producing food-grade *C. vulgaris* and *Nannochloropsis* [43].

#### New photobioreactor designs

More recently, several new photobioreactor designs have been reported. For example, Carone et al. [44] designed an alveolar flat panel photobioreactor; this reactor enhanced the CO_2_ bio-fixation rates using 1.3 cm thick alveolar flat-panels as light receptor. Gifuni et al. [45] developed an ultra-thin (3 mm) flat photobioreactor for increasing both biomass concentration and productivity, and maximum biomass productivity of 1.34 g m^−2^ h^−1^ was obtained with *C. sorokiniana*. Furthermore, a hybrid photobioreactor consisting of a bubble column reactor coupled to an illumination platform has recently been reported [46]. The reactor presented higher hydrodynamic performance (mixing time of 98 s), and biomass yield of 2.8 g L^−1^ was achieved in the reactor with *S. obliquus* CPCC05 [46]. However, scaling up of these systems may be difficult because of their complexity and potentially high cost.

#### Challenges of enclosed photobioreactors

Although higher biomass density can be maintained, the construction and maintenance costs of photobioreactors are ten times higher than those of open ponds, making them uncompetitive for the industrial production of microalgal biomass. Thus, photobioreactors can be used commercially to produce high-value bioactive substances, such as obtaining astaxanthin from *H. pluvialis*; it is foreseeable that these enclosed bioreactors will be continuously used to produce high-value products from microalgae in the future under aseptic conditions. Furthermore, there are several other problems, such as the accumulation of high concentration of O_2_ in the cultures and difficulties in cleaning. Until these problems are solved, the commercial application of enclosed reactors for microalgae will be challenging.

### High cell-density heterotrophic cultivation-fermenters

#### Characteristics of heterotrophic cultivation

Some microalgal species can grow in the dark or under light limitation, using organic carbon (e.g., acetate or glucose) as their sole carbon and energy source, a process known as heterotrophy. Heterotrophic cultivation in fermenters may result in higher cell productivity than that in open ponds and photobioreactors, as this growth mode eliminates the requirement for light. Therefore, this process may provide a cost-effective and large-scale alternative strategy for microalgal biomass production. Fermenters and photobioreactors represent enclosed systems that share many common features, such as pH and temperature control, and the progress in stirring and harvesting. The main differences between fermenters and photobioreactors include their energy source, oxygen supply, and sterility, which may lead to differences in the final biomass production of these two systems. Notably, high cell concentrations also mean the lower downstream process costs. Hence, the focus is now on heterotrophic cultivation of microalgae.

#### Development of heterotrophic cultivation

The key to heterotrophic production is that microalgal cultures must be axenic. This issue can be well-solved by drawing on proven techniques in microbial fermentation, for example, sterilization of fermenters and medium can be achieved using steam. Early attempts have been made to develop industrial production processes for microalgal heterotrophic cultivation. For example, studies on heterotrophic production of microalgae began in Japan in the late 1970s for *Chlorella*, and this process was applied to the industrial production of *Chlorella* in the mid-1990s. Subsequently, Cell Systems (Cambridge, UK) developed a process for the heterotrophic cultivation of *T. suecica* in 5000 L scale fermenters [47]. In addition to these heterotrophic batch processes, an industrial heterotrophic cultivation process for docosahexaenoic acid (DHA) production by *Cryptheconidium cohnii* was set up at Martek Biosciences in 1990s (Columbia, USA) [11].

During the last two decades, heterotrophic cultivation of microalgae has attracted attention. On one hand, high productivity of heterotrophic production has attracted the interest of microalgae enterprises. This process has been used to produce high-value products. For example, DSM (Heerlen, Netherlands) has commercialized the production of DHA and microalgal oil rich in DHA from two heterotrophic microalga, *Schizochytrium* sp. and *C. cohnii*, respectively, using a two-stage fed-batch process [48]. Duplaco (Oldenzaal, Netherlands) produces *Chlorella* for human nutrition using a proprietary process in which the microalgae are ‘fed’ with carbon source and grown in sterile fermenters; this process is expected to be expanded in the future to produce 1500 tons year^−1^ of *Chlorella* biomass. On the other hand, the heterotrophic cultivation processes are also being optimized constantly. Studies have reported increase in the yields of biomass and their by-products in *E. gracilis* and by *C. vulgaris* by optimizing complex medium composition and culture conditions, respectively [49, 50]. More recently, the ultrahigh‐cell‐density heterotrophic cultivation has been achieved in *C. sorokiniana* GT-1 and *S. acuminatus* using a fed-batch strategy; this process has successfully increased the biomass yield of *C. sorokiniana* GT-1 to 247 g L^−1^ and *S. acuminatus* to 283.5 g L^−1^ in 1000 L pilot-scale fermenters [19, 20]. Notably, a techno-economic analysis based on pilot-scale data showed that the cost of heterotrophic production of microalgal biomass is comparable to that of open systems if the biomass yield is higher than 200 g L^−1^ [20]. Ultrahigh‐cell‐density heterotrophic cultivation has also been studied for producing lutein from *C. sorokiniana* FZU60, and maximum lutein productivity of 82.50 mg L^−1^ day^−1^ was obtained using pulse-feeding with concentrated urea-N medium in a fed-batch culture [21]. These efforts further confirmed the commercial viability of producing microalgal biomass and high-value co-products using heterotrophic production processes.

#### Limitations of heterotrophic cultivation

Compared to photoautotrophic culture, heterotrophic production is limited by the lack of intracellular photosynthetically derived compounds. This may lead to the loss of the main advantage and practical application value of microalgae. Few attempts have been made to obtain high cell density with high cellular photosynthetic components. One potential route is mixotrophic culture of microalgae, but the contribution of this process to improving biomass production is limited [51]. Ogbonna et al. [52] first reported increase in chlorophyll and protein contents by transferring a highly concentrated microalgal culture from a 2.5 L fermenter to a photobioreactor, which confirmed the possibility that the nutrient composition of microalgae in heterotrophic production can be improved by supplementing light. SHDP was the first real large-scale process for improving photosynthetically derived compounds in heterotrophic cultivation; this process starts with obtaining high concentration of cells in heterotrophic culture, followed by dilution of the cultures to reduce the cell density, and finally photoinduction to increase the production of intracellular photosynthetic derivatives [53, 54]. Recently, SHDP has been used for commercial production of *Chlorella* and *E. gracilis* by Baoshan Zeyuan Co., Ltd (Yunnan, China).

Compared to open ponds and photoreactors, heterotrophic production is limited by the number of available heterotrophic algal species. However, as heterotrophic production may further increase productivities in the future after optimization of growth conditions, and owing to the ease in controlling the production process in heterotrophic production systems, heterotrophic cultivation of microalgae is expected to find worldwide application. In addition, the microalgal heterotrophic culture process is very similar to microbial culture technology; hence, it is possible to use proven microbial culture techniques and equipment to achieve heterotrophic mass culture of microalgae, which will considerably accelerate the industrialization of microalgae and their products.

## Microalgae harvesting: exploring suitable strategy for bulk biomass production

### Harvesting techniques of commercial microalgae

Microalgae harvesting is the process of recovering biomass from the culture medium. It represents one of the most important challenges for commercial-scale biomass production. Microalgae grow suspended in water; even the microalgal biomass in heterotrophic culture exceeds 20% dry weight with difficulty. Therefore, harvesting is energy and capital intensive, and can contribute approximately 30% of the total cost of microalgal production. The selection of harvesting methods varies with microalgae, mainly depending on the physiognomies of microalgae, cell density, and the value of the commercialized products from biomass. Currently microalgae harvesting involves mechanical, chemical, electrical, and biological methods. Conventional methods on a commercial scale involve centrifugation, filtration, and flocculation, which can be applied individually or in combination. However, these methods present some economic or technological disadvantages in the actual biomass production process. The comparison of various harvesting techniques is presented in Table 2.

**Table 2:** Comprehensive comparison of common harvesting processes in microalgal biomass production

Harvesting processes	Factors influencing feasibility	Recovery	Advantages	Disadvantages
Centrifugation				
Disc stack centrifuges	• Cell settling characteristics • Centrifugal force • Type of centrifuges	High	• Rapid and reliable • Suitable for almost all microalgal species • No chemicals	• High capital investment • Energy intensive • Cell damage by shear stress and high gravitational force
Scroll centrifuges		High		
Hydrocyclones		High		
Filtration				
Microfiltration	• Cell size • Flow rate • Transmembrane pressure difference • Turbulent flow	Low	• Less cell disruption • No chemicals • Simplicity of operating and functioning	• Fouling • High cost in filter membrane replacement and pumping • Suitable for large volume cells • Effective for low volume cultures • Low permeability and selectivity of membranes
Macrofiltration		Low		
Ultrafiltration		High		
Dead end filtration		High		
Vacuum filtration		High		
Pressure filtration		High		
Tangential flow filtration		High		
Flocculation				
Chemical flocculation	• Selection of cationic flocculants • Charge density • Electronegativity and solubility	High	• Low cost and high efficiency • Simple and fast • No energy input	• Presence of metal salt residues • pH dependent • Recycling of medium is limited
Electro-flocculation	• Selection of electrode materials • Current density • Electrolysis time • Composition of the microalgal suspension	High	• Non-species specific • No residual anions • Low chemical usage • Low power consumption	• Need for electrode replacement • Residual metals in algal biomass • pH changes • Temperature increase of algal suspensions and cell damage
Bio-flocculation	• Selection of bio-flocculants	General	• No chemicals or specific culture conditions are needed	• Highly species-dependent process • Unclear mechanism • Long flocculation period • Possibility of biological contamination
Auto-flocculation	• Changes in nitrogen, pH and dissolved oxygen	High	• No chemicals • Neutralizing negative charge	• pH dependent • Unclear mechanism • Unstable
Other processes				
Flotation	• Type of collector • pH and ionic strength • Type of bubble formation • Air tank pressure • Hydraulic retention time • Particle floating rate	High	• Short operation time • Low space requirement • Large scale harvesting • High flexibility with low initial cost	• Flocculant or surfactant required • Energy intensive • Ozoflotation is expensive
Gravity sedimentation	• Cell settling characteristics • Cytoplasmic density	Low	• Simple and low cost	• Time-consuming • Not reliable and effective

#### Centrifugation

Centrifugation is the oldest and most commonly used method of harvesting microalgae from their growth medium. The first known pilot-scale process of harvesting via centrifugation was reported in the early 1950s. Burlew [8] proposed to recover microalgal biomass from a large-scale culture unit using two centrifuges in series. The feasibility of harvesting via centrifugation depends considerably on the cell settling characteristics and types of centrifuges. Several centrifuges can be used for microalgae separation on industrial scale. These mainly include disc stack centrifuges, scroll centrifuges, and hydrocyclones [55]. Disc stack centrifuges are the most common industrial centrifuges, applying a force equivalent to 4000–14,000 times the force of gravity [56]. They can be used to concentrate microalgae with sizes between 3 and 30 μm [56], and are commonly used to recover high-value microalgae due to their high energy consumption. Bulk biomass harvesting requires centrifuges that can operate continuously. The scroll centrifuge may be the most promising centrifugal device for recovering microalgae, as they can be operated in continuous mode with high capacity and lower maintenance requirements; however, they are often limited by high capital cost and energy demand. Furthermore, the scroll centrifuge is not suitable for all types of microalgae, such as the commercially important *Chlorella* [57]. Hydrocyclone can also be operated continuously with a low maintenance requirement; its application in algae harvesting was first studied in 1980s and the results confirmed its poor reliability (only 0.4 solids) [58, 59]. Thus, hydrocyclone has been suggested to pre-concentrate algal biomass [58]. In addition to the high maintenance and energy costs, centrifugation also exposes algal biomass to high gravitational and shear forces, resulting in damage to the cell structure and loss of valuable materials [57].

#### Filtration

Filtration is a physical separation process; this technique allows fluid to flow through a membrane under gravity, pressure, or vacuum force, where microalgae can deposit on the membrane. Compared to other harvesting techniques, filtration can provide high-quality biomass because of low levels of cell disruption and the absence of chemicals in the membrane process. The main drawback of the process is fouling. This phenomenon often increases the flow resistance and reduces the filtration flux. Several filter designs have been developed depending on the hydrodynamic conditions and membrane characteristics [58]. Typical membrane materials mainly include polyvinylidene fluoride, polyether sulfone, polyethersulfone polyvinyl-pyrollidone, and polyvinyl chloride, as well as ceramic filtering layers [59]. Various methods, including ultrafiltration, microfiltration, macrofiltration, dead end filtration, vacuum filtration, pressure filtration, and tangential flow filtration were used for microalgae harvesting [59]. Filtration is efficient for harvesting microalgae with high cell volume, such as *Arthrospira* sp. and *Coelastrum* sp. However, microalgae with smaller cell sizes, such as *Chlorella* sp. and *Nannochloropsis* sp., tend to clog the filter membrane during filtration, resulting in reduced filtration efficiency. Tangential flow filtration provides a solution for smaller microalgae. It can somewhat alleviate the issue of filter fouling, as the medium flows tangentially across the membrane, maintaining the cells in suspension [60]. Filter membrane replacement and pumping are the main expenses associated with filtration. It is, therefore, effective for small volumes.

#### Flocculation

Flocculation represents a low-cost harvesting method; this process increases the particle size, reducing the energy requirement in dewatering process [61, 62]. Flocculation was first used in wastewater treatment and was investigated for microalgae harvesting using chitosan as the flocculant in the 1980s [63, 64]. It has been recognized as an excellent technique for harvesting microalgae, as it can be used on a large scale for various microalgal species [65]. Currently, three main processes have been extensively studied: chemical flocculation, physical flocculation (electro-flocculation) and bio-flocculation. Chemical flocculation, the most common method, usually uses cationic flocculants, such as metal salts (Al^3+^, Fe^3+^, Ca^2+^, and Mg^2+^) and macromolecule polymers (chitosan, polyacrylamide, and polyethyleneimine). The process causes aggregation of algal cells due to neutralization or reduction of the negative charge on the surface of the microalgae and/or due to the formation of bridging bonds. A study has shown that cationic polyelectrolytes are more effective than metal salts, with the ability to induce up to 35 times biomass concentrations [66]. Although economical, the chemicals used for flocculation can be hazardous and may contaminate the algal biomass. In particular, metal salts remain in the biomass residue after the lipids or carotenoids have been extracted. These metals may interfere with the use of the protein fraction of this residue as animal feed. Electro-flocculation is also used for algae harvesting. The technique uses an applied electric field to disrupt the electrostatic balance of individual microalgae, causing the algal cells to aggregate. Although the removal efficiency is high (80–95%), the process requires electrode replacement and maintenance, and metal residues may be present in the recovered biomass. Bio-flocculation is a promising method, as it does not require chemicals or specific culture conditions [59]. Bio-flocculation is assumed to be caused by extracellular polymeric substances (EPS) in the medium [67]. EPS can be produced by bacteria, microalgae, and fungi [59]. Therefore, flocculating species can be supplied to the algal growth medium to harvest microalgae. However, the mechanism underlying bio-flocculation is poorly understood. Some studies have suggested that bio-flocculation may be triggered by info-chemicals [68, 69]. The use of bacteria or fungi as flocculants is the principal drawback of bio-flocculation, as it leads to microbial contamination. This may also limit the use of microalgal biomass for food or feed applications. Thus, this technique is often used for wastewater treatment [70, 71].

#### Other techniques

Other techniques, such as flotation and gravity sedimentation, have also been developed for microalgae harvesting. Flotation involves introduction of air bubbles to transport the suspended matter to the top of the liquid surface, where it can be collected using a skimming process [59]. The technique is more effective than gravity sedimentation, especially for cultures with low density and self-floating properties. The main advantages are short operation time, low space requirement, and low initial equipment cost. The process is usually used after the flocculation process. However, the surfactants used for flotation may be toxic. Gravity sedimentation is a simple and inexpensive process, but disadvantages such as low efficiency and time-consuming limit its use in microalgae harvesting.

### Harvesting strategies for bulk biomass production

#### Harvesting strategies based on the final application

Currently, good harvesting methods are lacking, as the major drawbacks of each harvesting technique prevent them from being applied on a large scale in a non-toxic, cost-effective, or energy-efficient manner at the same time. Similarly, no single method or combination of harvesting methods appears to be suitable for all species. Nevertheless, thorough comparative analysis is required to determine the most appropriate harvesting method. These analyses should be based on some of the most critical factors in harvesting technique, such as recovery efficiency, concentration factor, biomass quantity and quality, cost, processing time, toxicity, and suitability for large-scale application [72, 73]. Considering that microalgae can be used in various applications and that different applications focus on different key criteria, the application of each microalgae should be analyzed specifically, i.e., the described parameters should be prioritized in a different order depending on the final application of the microalgal biomass [73].

**Fig. 3:**
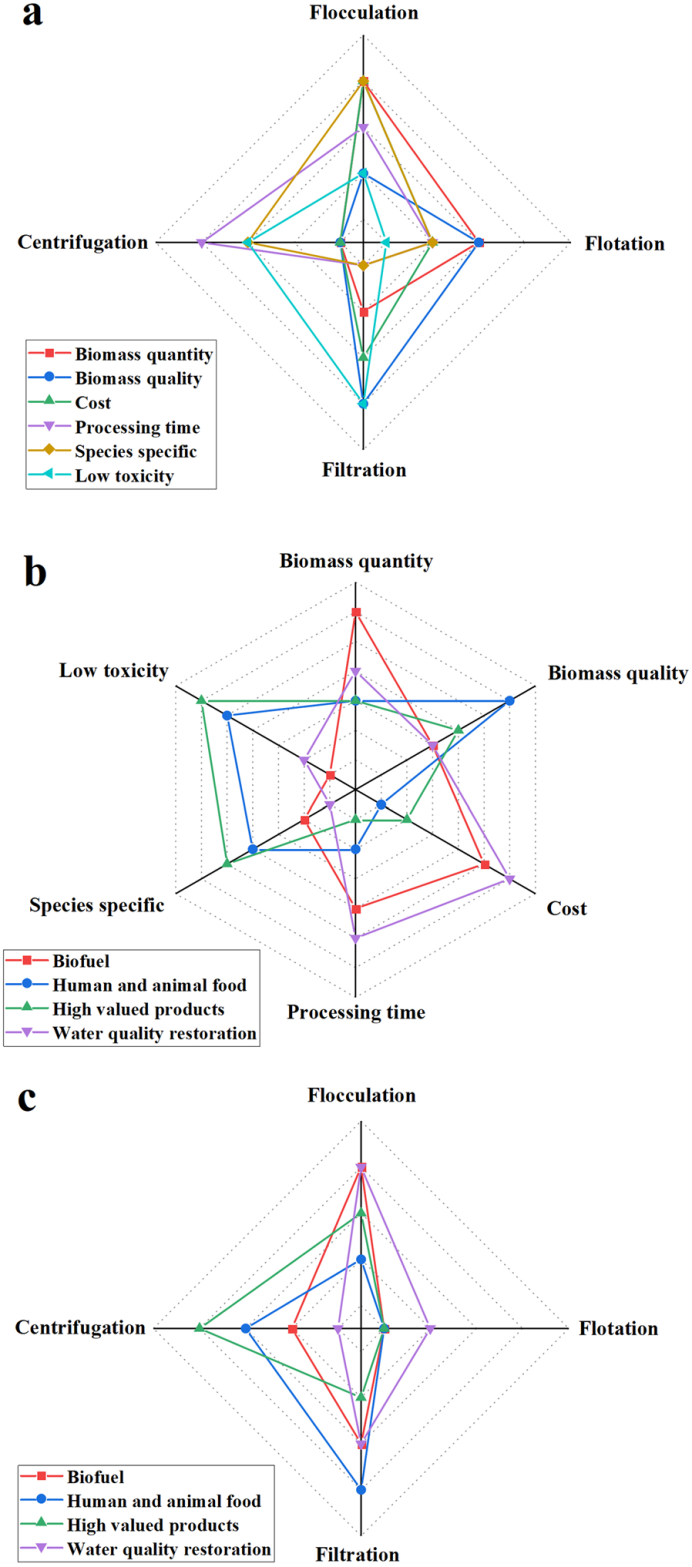
Comprehensive evaluation of optimal harvesting techniques for different applications. **a** Order of suitability of harvesting techniques for various criterions; **b** order of the most important criterions should be considered for various applications; **c** order for suitability of harvesting techniques for various applications

A possible approach that may be followed to determine the most appropriate method for each microalga should include the following: (i) the final application of the biomass recovered from microalgae should be clarified; (ii) the most important criteria should be considered for each application; (iii) the most satisfactory harvesting method for each criterion should be considered [59, 73]. Several studies have conducted similar analyses [59, 72, 73]. In these studies, six important criteria, including biomass quantity, biomass quality, cost, processing time, toxicity, and suitability for large-scale application, were used to assess the applicability of harvesting techniques to the main or potential applications of microalgal biomass. These applications include the production of human food, animal feed, high-value products, water quality restoration and biofuels. The most appropriate harvesting technology for each biomass application is selected by establishing a prioritized list of criteria for that application. Figure 3 summarizes the evaluation for harvesting techniques considering each criterion based on the main advantages and disadvantages of each harvesting method described in this study. The current demand for microalgal biomass is mainly for health food. In this regard, biomass quality, toxicity, and suitability for large-scale application are considered the key factors. This is also applicable for the production of animal feed. For high-value products, toxicity, biomass quality, and quantity are even more important. Algal biomass can be potentially used for making biofuels, and considering the current demand for low-cost biofuels, biomass quantity, cost and processing time are considered to be the most important criteria for biofuel production. Briefly, for industrial-scale production of microalgal biomass, flocculation, filtration, and centrifugation are the main options for harvesting. Centrifugation is the most suitable option for the production of high-value compounds due to its advantages in terms of biological quality, processing time, and suitability for large scale applications. In terms of biomass quality, filtration is considered to be the most suitable method for harvesting for human food and animal feed. Considering the cost requirements, flocculation appears to be the best option for wastewater treatment and biofuel production.

#### Two-step harvesting process

In many cases, the use of a combination of two or more harvesting methods can lead to further improvements in harvesting efficiency, production cost, and processing time [74]. A typical combination of harvesting techniques includes a pre-concentration/concentration step, followed by dewatering. In a two-step process, the microalgal suspension from the culture system is first concentrated to an algal slurry with 2–7% total suspended solids; then, in a second step, the slurry is dewatered to 15–25% TSS [59, 75]. The processes used for the first step of concentration include flocculation, sedimentation, flotation and electro-assisted technique. Centrifugation and filtration are usually used for the final dewatering process. This step is more expensive as it requires a higher energy input than the thickening process. Several studies have reported the advantages of using different combinations of harvesting methods. For example, Hapońska et al. [76] evaluated the application of pH-induced sedimentation combined with dynamic filtration for microalgal dewatering at a pilot scale. High concentration factors of 207.4 for *D. tertiolecta* and 245.3 for *C. sorokiana* were achieved using a combination of these two techniques. More recently, Min et al. [75] used the resonance vibration submerged membrane system as a pre-treatment process prior to centrifugation for concentrating *C. vulgaris*; the system was evaluated and was found to be less energy intensive than conventional systems. Thus, these studies proved the potential benefits of using multiple methods for microalgae harvesting in terms of recovery efficiency, processing time, and process economics.

## Microalgae drying: balancing cost and quality

### Drying techniques of commercial microalgae

Drying is usually as the last harvesting step. This process requires the removal of moisture to ≤ 12% to obtain dry microalgal biomass for downstream product production. The dried microalgae are easy to store and transport, as well as to use in bio-refinery and in the food and feed industry. Drying also represents a significant fraction of the total production costs. Since the mass cultivation of microalgae, several drying techniques have been developed. The commercial techniques mainly include (i) solar drying, (ii) convective drying, (iii) spray drying, and (iv) freeze drying. The different processes have their own distinctive features. The selection of drying method is critical for the subsequent processing and quality of the final products.

#### Solar drying

Solar drying, the most traditional and cost-effective method for microalgal powder production, has been used for hundreds of years to stabilize the moist algal biomass. In some open processes, the heat for water evaporation is provided by solar radiation and moisture removal by natural air currents. This may be time-consuming, and a large drying surface and the efficiency of the process is directly dependent on the weather conditions. Moreover, longer processing times and exposure to open environments may increase the risk of spoilage or development of off-flavors. Several strategies and facilities have been developed to address these issues. Some closed solar dryers can reduce the moisture content of the final product to less than 10% within 5 h of drying and remain low energy and exergy efficient [77, 78]. These dryers usually consist of a solar heater, a drying chamber, and an airflow system. Although the process can further improve the quality of the algal powder, research on this is negligibhas focused on solar dryers for microalgae.

#### Convective drying

Convective drying is popularly used for drying microalgae. It is performed in a type of convective hot air dryer and is commonly used in small-scale production. It usually includes draft oven drying, convective tray drying, microwave oven drying, convective tunnel drying, and continuous conveyor belt drying [79–83]. Several studies have evaluated the potential for large-scale application of these processes. For example, Chen et al. [84] assessed the effect of heating rate on the pyrolysis of *C. vulgaris* and measured energy consumption using microwave drying. A study indicated the strong influence of process temperature on chlorophyll a content and hue angle (relative to sample color) under the same conditions of convective drying, with a sharp reduction in chlorophyll concentration at drying temperatures up to 40 °C [85]. This fact was also confirmed by Oliveira et al. [86] who evaluated the effect of drying temperature on the functional components of *Arthrospira*; results showed that convection drying temperatures above 45 °C could cause phycocyanin degradation. Therefore, the optimization of convective drying conditions is important for pilot-scale applications. Moreover, studies are required to minimize energy consumption.

#### Spray drying

On a commercial scale, spray drying is the most commonly applied method. This technique was first proposed in the early 1950s for the production of microalgal powder [8]. Spray drying involves the atomization of the algal slurry to produce droplets, which are dried into individual particles while moving through the hot air. Although the algal slurry is exposed to higher temperatures in a shorter period of time, the drying of single droplets provides a large surface area per unit volume of liquid, which facilitates rapid drying and reduces degradation of product quality. Therefore, this process is the preferred method for drying high value microalgae products [87]. For example, spray drying of *D. salina* biomass produces powders with very low degradation rates of β-carotene and its isomers [88]. Green dark or green microalgal powder could be produced by optimizing the process conditions [87]. The main factors affecting the quality of the dried product include droplet size, air temperature, liquid flow rate, surface tension, density and viscosity of the algal slurry should also be considered. Studies have shown that the morphology and color of the microalgal powder is highly dependent on the spray drying process and temperature [89]. Volatile compounds are potentially lost in this method. The shelf life of compounds can be increased by mixing algal slurry with an encapsulant to produce microcapsules [90, 91]. High installation and energy/operation costs also make low-value products economically unviable.

#### Freeze drying

Freeze drying is another common drying technique used in the food industry. This is a two-step process. The algal slurry is first frozen and transferred into a vacuum chamber, which then provides heat for water sublimation (latent heat of sublimation) via radiation or conduction (hot plate) [92]. Similar to spray drying, it is mainly used for processing high added value products and foods, although the loss of nutrients at high temperatures is avoided. Freeze drying has been reported to preserve most of the protein in the dried microalgae biomass, with protein losses of less than 10% [93]. Ahmed et al. [94] investigated the effect of different drying processes and storage methods on the astaxanthin concentration of dehydrated *H. pluvialis* powder. As expected, the freeze-dried biomass retained higher amount (~ 30%) of astaxanthin than the spray-dried biomass. The stability of freeze-dried algal biomass may be affected by its extremely high porosity [95]. This may accelerate the oxidation of lipids and pigments. Therefore, to maintain the high quality of the product, the vacuum packaging should be considered when storing freeze-dried powders. In addition, small changes in the operational factors of freeze drying may significantly impact the efficiency of cell disruption [96]. For example, when samples are frozen slowly, larger intracellular ice crystals can form, causing cell wall disruption [97]. In contrast, the high installation and operation costs of industrial-scale equipment limit the application of these processes to low-value products.

### Finding suitable drying processes for high-quality biomass production

**Fig. 4:**
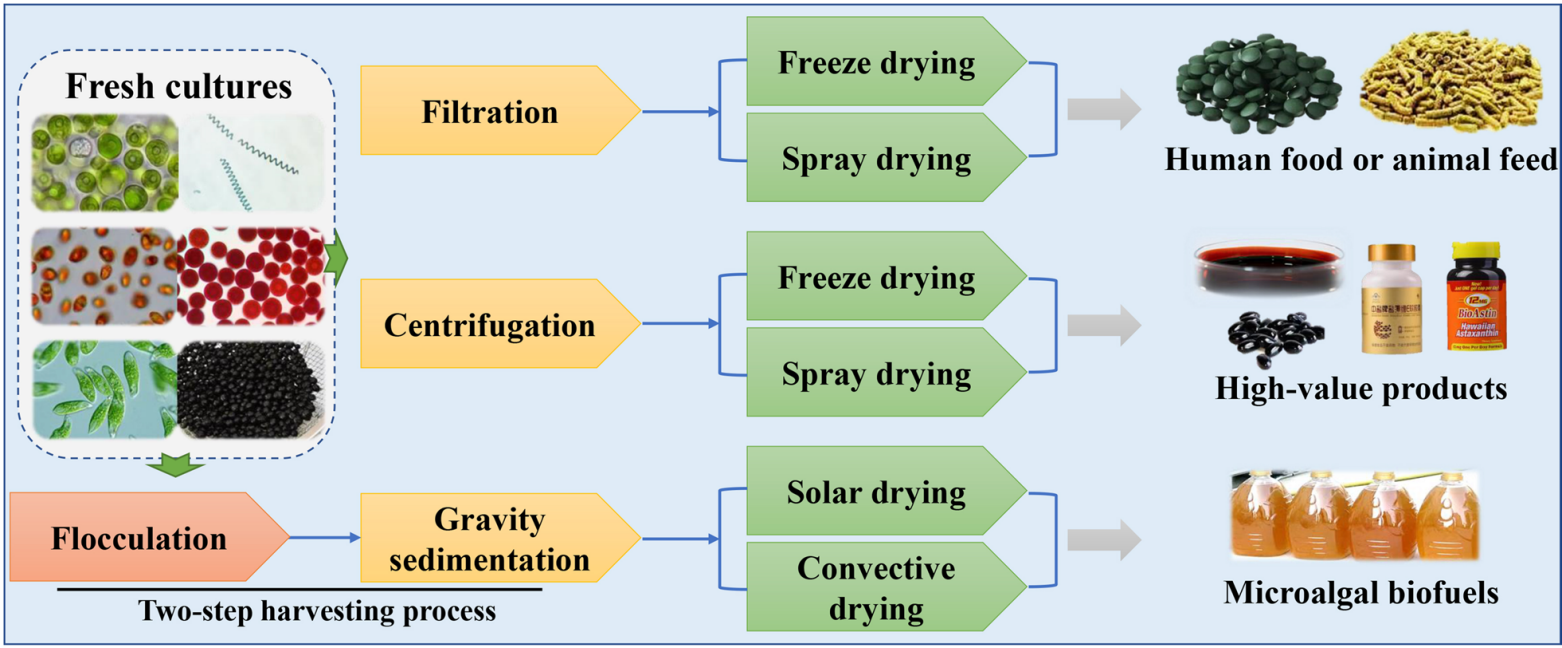
Processes of microalgae harvesting and drying for different end products

Drying is also an energy and capital-intensive process [98]. The degree of dryness of the algal biomass obtained using various drying techniques is close. In most cases, energy efficiency and process engineering are focused on at the expense of the quality of the product. In particular, little attention has been paid to the effect of dehydration on the functional and nutritional composition of the final products [99]. Similar to microalgae harvesting, selection of the drying method is also highly dependent on the final application of the biomass and the acceptable cost of producing the target products. The processes of microalgae dewatering and drying for some different end products is shown in Fig. 4. Among the methods studied and applied for producing algal biomass for human use, spray drying and freeze drying have been used most widely [100–102]. This is because microalgae contain valuable compounds, such as phycocyanin, lutein, β-carotene, and astaxanthin, which are easily destroyed or degraded by heat, light, or oxidation [100–102]. In such cases, it is necessary to use more delicate (and often more expensive) drying techniques to process biomass for high-value products. Spray drying is usually preferred, because it is suitable for large producers. However, freeze drying can overcome the shortcomings of spray drying in terms of loss of functional composition under high temperature or inappropriate storage conditions [94]. For some compounds that can tolerate higher temperatures, such as EPA and DHA, spray drying or convective drying can be used to reduce operating costs. However, when microalgae are used for biofuel production and fermentation, low-cost drying methods (e.g., solar drying and convective drying) are often chosen [100]. Notably, all drying methods used should be optimized to avoid spoilage of the microalgal biomass or inhibition of downstream processing.

In summary, further research is still required to improve not only the drying methods, but also to analyze degradation during storage, especially with respect to the sensitivity of dried microalgal biomass to light, heat, and oxygen, which will ensure supply of high-quality products to consumers. Although many new drying techniques have been developed in recent years, which may be based on the same principles with only some modifications to parameters and equipment, studies on quality assessment for drying microalgae are not common in literature. Different drying methods applied to commercial species with interest in evaluating quality characteristics are summarized in Table 3.

**Table 3:** Different drying methods applied to commercial species with interest in evaluating quality characteristics

Species	Drying methods	Drying conditions	Quality assessment	Conclusions	References
*Arthrospira* sp.	Convective drying	Temperature: 70 °C; Time: 8 h	• Total protein analysis • Phycobiliprotein analysis	• Convection may be the most appropriate way to have food grade feedstock • The phycobiliprotein fractions are greatly affected by the drying method	[98]
	Freeze drying	Primary drying at − 30 °C for 6 h; secondary drying at − 52 °C for 48 h			
	Spray drying	Inlet air temperature: 180 °C; feed rate: 2.16 kg h^−1^			
*Chlorella* sp.	Convective drying	Temperature: 40–140 °C	• Chemical composition • Colour characterization • Surface structure analysis	• *Chlorella* should be dried at 60–80 °C • The dominant mechanism in *Chlorella* drying is diffusion	[99]
	Freeze drying	Temperature: − 50 °C; time: 24 h	• Protein analysis • Elemental composition • Lipid content analysis • Total chlorophylls analysis	• Freeze drying biomass provide the highest lipid content (10.7%) and total chlorophylls (204.6 µg mL^−1^) • The free fatty acids in the extract from solar drying biomass were highest	[100]
	Solar drying	Temperature: 25–58 °C; time: 72 h			
	Spray drying	Inlet air temperature: 170–190 °C; outlet air temperature: 95.0 °C; feed rate: 7.00–9.00 mL min^−1^; encapsulants: maltodextrin	• Total carotenoid analysis • Moisture content and water activity • Colour properties • Drying efficiencies	• The moisture, total carotenoid, and chlorophyll-a contents were modelled significantly • The use of encapsulants in spray drying for food applications is essential	[101]
*D. salina*	Spray drying	Inlet air temperature: 120 °C and 140 °C; outlet air temperature: 95.0 °C; Feed rate: 400 mL min^−1^ and 600 mL min^−1^; encapsulants: maltodextrin, gum Arabic, gelatin	• Chlorophyll a content analysis • β-Carotene content analysis	• Microcapsules composed of maltodextrin: gum Arabic (90:10) exhibited the highest capability (93.22%) to preserve the β-carotene	[102]
*H. pluvialis*	Freeze drying	Temperature: − 40 °C; time: 16 h	• Astaxanthin content analysis • Moisture content analysis	• Freeze–drying led to 41% higher astaxanthin recovery • Freeze–drying followed by vacuum-packed storage at − 20 °C can generate AUD$ 600 higher profit	[94]
	Spray drying	Inlet air temperature: 180 °C; outlet air temperature: 110 °C			
	Spray drying	Inlet air temperature: 180 °C; Outlet air temperature: 80 °C; Encapsulants: maltodextrin and gelatin (2.1:1)	• Astaxanthin content analysis • Microcapsule powder analysis	• Microencapsulation yield reached 38.02% and the highest encapsulation efficiency was 71.76% • Astaxanthin microcapsules could be applied in the food industry	[103]
*E. gracilis*	Spray drying	Inlet air temperature: 155 °C; Outlet air temperature: 95 °C	–	• The dried powder could be used directly in many *Euglena* powder products	[24]
*N. sphaeroides*	Pulse-spouted microwave freeze drying	Temperature: − 45 °C; pulse frequency: 3 times h^−1^; pulse time: 0.3 s	• Colour properties • Texture • Flavour analysis • Ascorbic acid analysis • Antioxidant capacity	• Biomass by pulse-spouted microwave freeze drying has higher antioxidant activity, and has the advantages of short drying time and low energy consumption than freeze drying and convective drying	[104]
	Freeze drying	Temperature: − 45 °C			
	Convective drying	Temperature: − 60 °C			

## Control of biological contaminants

### Transmission routes of biological contaminants

The ideal state for microalgal mass cultivation is a production system in which only target microalgae are growing. However, biological contaminants will inevitably enter the cultures, both in open ponds or photobioreactors, which are relatively open systems that require the transfer and exchange of gases between the culture system and the external environment, in addition to the water input. In particular, microporous membrane filtration does not remove viruses from the air, and large volumes of water cannot be treated using conventional microbial fermentation with thermal sterilization. These limitations in production systems are the main cause of the spread of biological contaminants. Therefore, responsive strategies must be adopted to avoid them during mass cultivation of microalgae.

### Species and their contamination mechanisms

So far, many biological pollutants have been reported. These include zooplankton, bacteria, viruses, and other microalgae. The common biological contaminant species and their contamination mechanisms are summarized in Table 4.

**Table 4:** Some biological contaminants and their contamination modes for commercially important microalgae

Species	Target algae	Contamination mode	Algicidal activity	References
Zooplankton				
*Brachionus plicatilis*, *Euplaesiobystra hypersalinica*	*A. platensis*	Grazing	n/d	[105]
*Brachionus plicatilis*, *Frontonia* sp.	*A. platensis*		n/d	[106]
*Brachionus rubens*	*C. sorokiniana*		99.8%	[107]
*Poterioochromonas malhamensis*, *Vannella* sp.	*C. sorokiniana*		38–59%	[108]
*Brachionus calyciflorus*	*C. vulgaris*		n/d	[109]
*Pseudobodo* sp. KD51	*C. vulgaris*		n/d	[110]
*Naegleria* sp., *Cladotricha* sp.	*D. salina*		n/d	[111]
Bacteria				
*Bacillus fusiformis*	*Chlorella* sp.	Secreted metabolites	45.6% (1 d)	[112]
*Enterobacter cloacae*, *Gibberella moniliformis*	*C. pyrenoidosa*	n/d	n/d	[113]
*Bowmanella denitrificans*	*C. vulgaris*	Secreted metabolites	28.7%	[114]
*Bacillus thuringiensis* ITRI-G1	*C. vulgaris*	Secreted AES-Bt agents	100% (8 h)	[115]
*Microbacterium paraoxydans*	*C. vulgaris*	Secreted atrazine-desethyl	64.38%	[116]
*Ponticoccus* sp. CBA02	*D. salina*	Secreted metabolites	n/d	[117]
*Sagittula stellata*	*D. salina*	n/d	52.4% (6 d)	[118]
*Paenibacillus polymyxa* MEZ6	*H. pluvialis*	Secreted metabolites	46.3%	[119]
Other algae				
*Oocystis* sp.	*Arthrospira* sp.	Resource competition	n/d	[120]
*Coelastrella* sp.	*H. pluvialis*	Resource competition	n/d	[121]
*Scenedesmus* spp.	*H. pluvialis*	Resource competition	n/d	[122]
Virus				
XW01	*Chlorella* sp.	Infection	n/d	[123]
ATCV-1	*Chlorella* sp.		n/d	[124]
OSy-NE5	*Chlorella* sp.		n/d	[125]
PBCV-1	*Chlorella* spp.		n/d	[126]

#### Zooplanktons

Zooplanktons are the main cause of culture failure. They usually act as predators of microalgae, reducing algal concentration and production to low levels in few days. The common predatory species in the microalgal mass cultivation are ciliate [127], rotifer [109], cladocera [128], and copepod [129]. A study has reported that the presence of *Brachionus rubens* reduced the biomass production of *C. sorokiniana* by up to 99.8%, leading to the collapse of the algal culture [107]. Similarly, another report confirmed that the outbreaks of the cladoceran, Daphnia, in open algal ponds could reduce the dry weight of common microalgal strains by 12.5–87.87% [130]. Zooplanktons follow two feeding mechanisms: mechanical (negative) and behavioral (positive) [131]. Factors such as temperature, light, and food availability influence zooplankton feeding, particularly for copepods, which select negative feeding mechanisms when food concentrations are low, and positive feeding mechanisms when food densities exceed a critical value [129].

#### Bacteria

Some bacteria, called phytoplankton-lytic bacteria, can also inhibit the growth of microalgae. Most known algicidal bacteria belong to Bacteroidetes/Cytophaga/Flavobacterium (55%) and γ-Proteobacteria (45%), and others (5%) belong to the Gram-positive genera, *Micrococcus*, *Bacillus*, and *Planomicrobium* [132]. They lyse microalgal cells via direct attack or indirect attack mediated by secreted extracellular compounds. Only few phytoplankton-lytic bacteria tend to attack directly. Commonly studied direct-attack species include *Xanthus* sp., *Saprospira* sp., and *Pseudoalteromonas* sp. Most phytoplankton-lytic bacteria prefer to attack indirectly. For example, *Vibrio* sp. have been reported to lyse algae by producing extracellular alga-lysing substances such as β-cyano-l-alanine and some unknown non-proteinaceous substances [132, 133]. Moreover, *Bacillus* sp. SY-1 has been shown to lyse a harmful dinoflagellate by secreting a novel alginate [134]. A similar study was conducted by Liao and Liu [135]; the results demonstrated that metabolites secreted by *B. fusiformis* possess algicidal activity against a wide range of microalgae. Although many algicidal bacteria have been identified so far, further research regarding the algicidal mechanisms of new species and the development of appropriate prevention and control strategies are required.

#### Virus

Viral infection also significantly reduces the concentration of microalgal cells in open ponds within a few days, and their mechanism for infecting living cells is well-known. The short replication cycle and host specificity of viruses suggest that they can rapidly reduce microalgal amounts or cause exceptionally low growth rates. Both prokaryotic cyanobacteria and eukaryotic algae can be infected by viruses [123, 136]. A virus that can infect cyanobacteria (LPP virus) was first reported in the 1960s [136]. This cyanobacterial virus could infect several hosts, including *Lynbya* sp., *Phormidium* sp., and *Plectonema* sp. Subsequently, the first eukaryotic algal virus, CCV virus, was also identified, with specificity for *Chara coralline* [137]. However, algal viruses and control measures for such organisms remain largely unexplored [125, 126]. Owing to the limited knowledge regarding algal viruses, recommendations for microalgal mass culture cannot be made immediately.

#### Other microalgae

In addition to the above biological contaminants, other microalgae can also inhibit the growth of target microalgae [121, 122]. These contaminants have attracted attention because of quality control issues and their toxic effects on the environment and animals/humans. Resource competition and allelopathy are main mechanisms via which a target is contaminated by other strains. The former indicates that unwanted photosynthetic species will outgrow the target microalgae, and/or compete for available resources. Microalgal allelopathy is a phenomenon in which photosynthetic species release antagonistic chemicals for inhibiting the growth of target microalgae [138]. For example, chlorellin, released by *C. vulgaris*, may significantly inhibit the growth of *Pseudokirchneriella subcapitata* [139]. These substances mainly include polyunsaturated fatty acids and their derivatives, alkaloids, and microcystins [138]. Contamination of other microalgae can, therefore, pose a serious safety threat, as toxins released into the cultures can be consumed by animals or humans after biomass harvesting.

### Strategies for controlling biological contaminants

Contamination of microalgal cultures is one of the main obstacles currently hindering the development of microalgal biotechnology. Cost-effective strategies must, therefore, be developed to control unpredictable biological contaminants, as the loss of even a single algal reactor in an array can significantly affect the productivity and yield of the entire facility. Several measures have been developed, including filtration, the use of chemical additives, and changes in the environmental conditions [129]. The use of these measures varies according to the pollutant and in some cases could be combined with several other strategies to control biological pollutants. Current defenses continue to focus on controlling zooplanktons as they rapidly consume algae, significantly reducing biomass production, and are thought to be responsible for harmful algal blooms.

#### Filtration for controlling larger contaminants

Filtration represents a physical method. Unlike microalgal harvesting, this method for controlling contaminants allows the microalgae to flow through the netting, while the larger biological contaminants remain on the netting. Filtration is considered an effective method of removing larger organisms, such as rotifers and copepods, but not smaller rotifer eggs and developing young individuals [129]. Therefore, for complete removal of macrobiotic contaminants, the microalgal solution should be filtered continuously for 3–4 days. In addition, the resistance of different algal strains to zooplanktons varies. Some algal populations have successfully resisted grazing pressure, such as *Chlorella* spp. and *Tetreselmis* spp. [140]. A potential strategy involves using high resistance strains for open cultivation wherever possible. In contrast, algal strains with weak or no resistance to grazing are cultivated using enclosed systems or alternative species. Considering that the higher cost of filtration only allows its use on a small scale, the above strategy appears to be more feasible.

#### Chemical control

Chemical control is a potentially viable method for eliminating biological contaminants. Many studies have reported chemical treatments for controlling biological contamination in open microalgal cultures. For example, Moreno-Garrido and Cañavate [141] reported that 10 mg L^−1^ quinine was effective in killing ciliates, with less damage to *D. salina* cells. In addition, the use of ammonium bicarbonate in culture can control rotifers and cladocerans, and can also provide an additional source of nitrogen and carbon at low cost [142]. However, the effectiveness of ammonium bicarbonate in controlling zooplankton contamination is significantly reduced at high temperatures as the ammonia evaporates [143]. An effective, safe, and low-cost method of controlling biological contamination is required. Botanical pesticides have been proposed as a potential control agent for zooplanktons in microalgal mass cultivation [144]. Various botanical pesticides, such as celangulin, matrine, azadirachtin, and toosendanin, are all being considered for use as biological control agents in microalgal mass cultivation [144]. For example, toosendanin has been shown to be effective for rotifer and ciliate contamination control [143, 144]. Considering its relative safety toward microalgal cells and low cost, the use of toosendanin for zooplankton control in microalgal mass culture appears promising. The use of chemical agents must be considered in terms of their impact on the final application of microalgal biomass, especially when it is used as animal and human food. In summary, the development of biopharmaceuticals that do not produce chemical residues without damaging the target microalgae may be more promising.

#### Changes in environmental conditions

Changes in certain environmental conditions, such as temperature and pH, can also be used to control biological contamination. This strategy depends on the survival conditions of the target microalgae and/or contaminants. Several studies have confirmed the effectiveness of this approach. For example, Hallegraeff et al. [145] confirmed that *Gymnodinium catenatum* and *Alexandrium catenella* can be easily killed using temperatures as low as 35 °C and 38 °C, respectively. This has potential application in the treatment of input water for algal cultivation. Adjustment of the pH of cultures is commonly used for killing or removing of biological contaminants [129, 146]. Becher [147] recommends lowering the pH to 3.0 for 1–2 h to control rotifers. Moreover, the amounts of contaminants in the biomass can also be limited by controlling the nutrient composition of the medium to maintain the target microalgal population. For example, harmful algal growth increases when diatom populations are starved by low silica levels; thus, managing silica levels is key to ensure that diatoms grow faster than other species [148].

#### Research needs for control of biological contamination

The control of biological contaminants is essential for the production of sufficient and high-quality microalgal biomass. However, a number of key issues still need to be addressed. For example, the application of control strategies also requires comprehensive understanding of the range of adaptation of target microalgae and biological contaminants to ecological factors. Bacterial contamination in open ponds is inevitable; hence, methods of reducing harmful bacteria and increasing beneficial bacteria (e.g., nitrogen fixing bacteria) is the focus of future research. Moreover, research on algal lysing viruses should be intensified, as current knowledge regarding algal viruses is limited. The other caveats regarding the control of algal toxins in biomass production include the lack of standards for acceptable levels of toxicity in algal biomass or compound feeds. In addition, the development of sensors for monitoring various biological contaminants is also necessary.

## Biotechnologically gifted strains of microalgae

In microalgal biotechnology, suitable species can be grown as production strains in aquaculture. Although tens of thousands of microalgae exist in nature, only a few gifted strains are used for commercial biomass production. In particular, over the past decade, the bulk of annual biomass production is dominated by six species, namely, the cyanobacteria, *Arthrospira* and *Nostoc* (cultivated only in China), the green microalgae, *Chlorella*, *Dunaliella*, and *Haematococcus*, and the flagellate, *Euglena*. Table 5 lists the gifts, bottlenecks, and technologies for further improving biomass production of these species.

**Table 5:** Gifts, bottlenecks, and key technologies in biomass production of some important commercial microalgae

Species	Gifts	Bottlenecks in mass production	Cultivation mode	Key techniques	Yield (tons)	References
*Arthrospira* sp.	• High growth rate • Grown in alkaline conditions	• High NaHCO_3_ consumption • High growth temperature requirements	• Autotrophic	• CO_2_ replenishment technology in raceway ponds • Breeding techniques for low temperature tolerant strains	12,000	[25]
*Chlorella* sp.	• High growth rate • Multitrophic mode	• Low level of photosynthetically derived compounds in heterotrophic mode	• Autotrophic • Heterotrophic	• High cell-density heterotrophic cultivation process • SHDP process	5000	[26]
*D. salina*	• High β-carotene content under stress conditions • Grown in high salinity conditions	• High medium costs • *D. salina* cells are fragile and difficult in harvesting	• Autotrophic	• Salt-making mother liquor or natural seawater used as medium for *D. salina* culture • Flotation process used for *D. salina* harvesting • Two-step cultivation process	1200	[27]
*H. pluvialis*	• High astaxanthin content under stress conditions	• Sensitive to biological contaminants • Astaxanthin is easy to be oxidized	• Autotrophic	• Two-step cultivation process • Microencapsulation process for *H. pluvialis* powder and astaxanthin	800	[28]
*E. gracilis*	• High growth rate • Multitrophic mode • Low pH tolerance	• Low level of photosynthetically derived compounds in heterotrophic mode	• Autotrophic • Heterotrophic	• SHDP process	70	[29, 30]
*N. sphaeroides*	• Cell population growth	• Sensitive to biological contaminants • High requirements for aquaculture water quality	• Autotrophic	• Breeding techniques for high quality strains • Water treatment technology for water hardness reduction	200 (fresh weight)	[6]

### *Arthrospira*

**Fig. 5:**
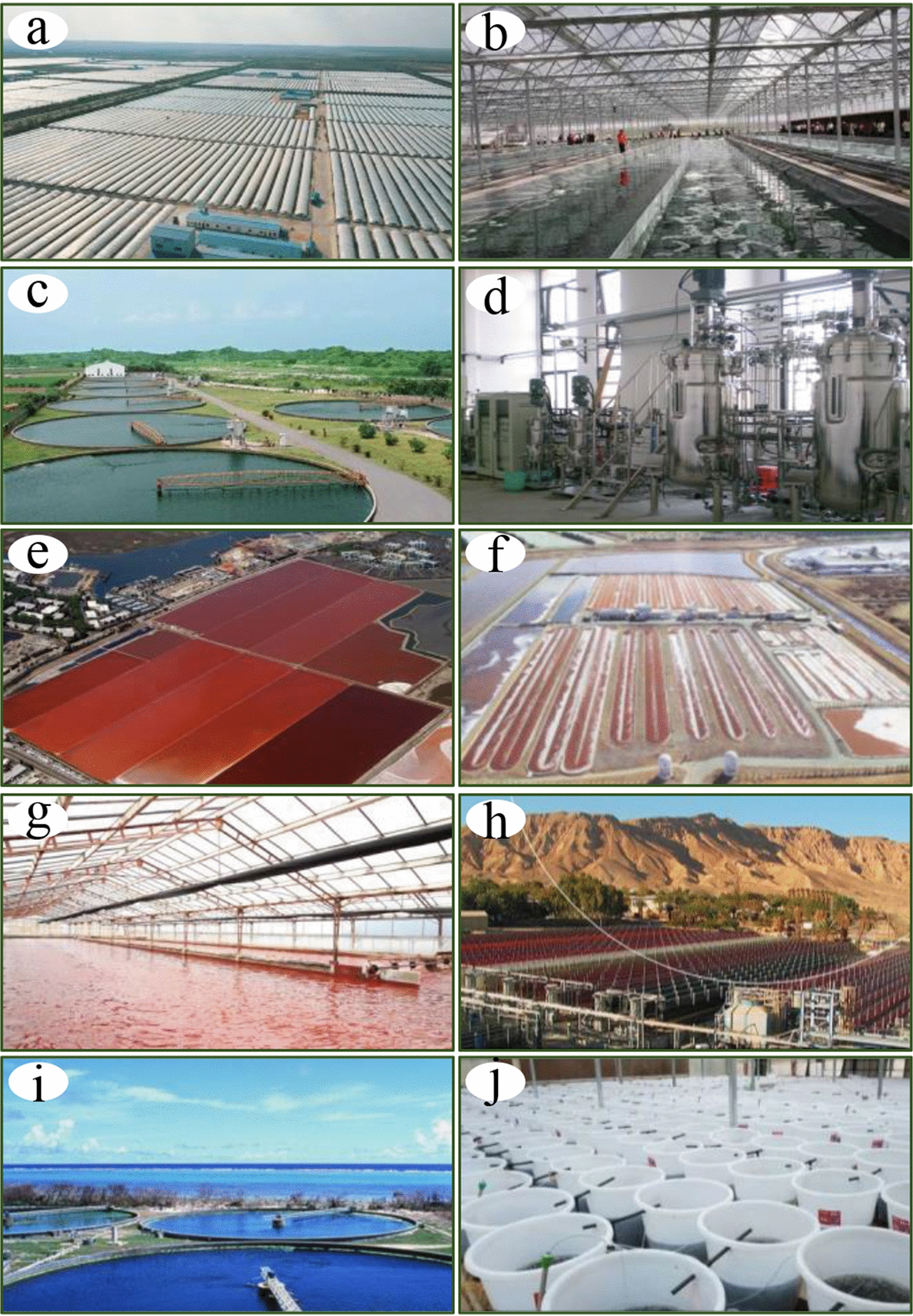
Examples of various biomass production systems for cultivation of commercial microalgae. **a**, **b** cultivation facilities (open raceways in greenhouse) of *Arthrospira* in Erdos (China); **c** circular ponds for *Chlorella* cultivation (Sun *Chlorella*, Japan); **d** heterotrophic culture facilities for *Chlorella*; **e** cultivation of *D. salina* at Cargill lakes in San Francisco Bay (USA); **f** *D. salina* cultivation using open raceway ponds by NBT Co., Ltd. (Eilat, Israel); **g** open raceways in greenhouse for *H. pluvialis* cultivation by Green-A (Yunnan, China); **h** tubular photobioreactors used for *H. pluvialis* cultivation by Algatech (Israel); **i** *E. gracilis* cultivation using circular ponds by Euglena Co., Ltd. (Japan); **j** a demonstration site for the indoor cultivation of *N. sphaeroides* in Plateau algal Research Center (China)

*Arthrospira* (Cyanophyta) is a multicellular filamentous cyanobacterium that grows naturally in subtropical alkaline lakes with an optimum temperature of approximately 35 °C. It represents the most successful commercially available microalga. In productive cultures, two species, *A. platensis* and *A. maxima*, were widely cultivated in open raceways or tubular photobioreactors. The first commercial production started in the 1970s in Mexico. Currently, *Arthrospira* is produced in over twenty countries. The mass cultivation of this cyanobacterium contributes to over 50% of the global microalgal production, with total annual production estimated at about 12,000 metric tons [25]. China, in particular, produces more than 60% of the world’s *Arthrospira* biomass, thanks to improvements in raceway ponds (Fig. 5a, b) and the breeding of low-temperature tolerant species. Recent statistics showed that 8327 metric tons of *Arthrospira* were produced in China in 2021, and 8828 metric tons are expected to be produced in 2022.

*Arthrospira* biomass is mainly used as human food, animal feed, and source of certain chemicals. In addition, mass cultivation of this alga has also been attempted for sewage treatment. The challenges for the *Arthrospira* industry are monotonous application markets and unclear market positioning. In addition to the food and nutraceutical sectors, the *Arthrospira* industry needs to develop other new application markets to increase the resilience of the industry. Much remains to be elucidated about the pharmacological activities and mechanisms of action of *Arthrospira*, particularly in the areas of antioxidant and antitumor activities; therefore, the key to future medical research is to develop the technology for isolation and purification of antioxidant and antitumor active substances. The cost of biomass production and the quality of the product still do not meet the demands of the market, which needs to be addressed through technological innovation and improvement of standards. Reducing the loss of nutrients during processing or obtaining fresh *Arthrospira* as dietary supplement by combining with the rapidly developing Internet of Things is another direction for the development and extension of this industry chain. Briefly, the various processes in the value chain of industrial production of *Arthrospira* all pose significant challenges.

### *Chlorella*

*Chlorella* (Chlorophyta), a genus of unicellular green microalgae living in freshwater, seawater, and terrestrial habitats [2], was the first species to be used commercially for biomass production. Commercial species mainly include *C. vulgaris* and *C. pyrenoidosa*. Currently, it is usually cultivated phototrophically in open ponds, cascades, and enclosed tubulars, as its high growth rate prevents contamination by other microalgae. A circular pond is the most common device for commercial biomass production of *Chlorella* (Fig. 5c). It can also grow under mixotrophic and heterotrophic conditions with the addition of acetic acid and glucose (Fig. 5d). Countries and regions where commercial production has been achieved include Japan (companies, such as Sun *Chlorella* and Yaeyama), mainland China (King Dnarmsa and C.B.N Microalgae, etc.), China Taiwan (*Chlorella* Manufacturing and Far East Bio-Tech, etc.), Korea (Daesang), Germany (Algomed), and Portugal, with total annual biomass production of about 5000 metric tons [26].

The success of mass cultivation of this microalga photoautotrophically, heterotrophically, and mixotrophically has led to a stable *Chlorella* industry for human nutrition and animal feed due to its high nutrient content. In recent years, the mass cultivation of *Chlorella* has also shown its potential for applications, such as bioremediation, biofuel production, and as a raw material for biofertilizers. However, the current production systems and processes of *Chlorella* are neither cost-effective nor energy-efficient, rendering these potential applications impractical. In particular, the cell processing requires both effective and efficient harvesting and mechanical disruption of cellulose cell walls. Breakthroughs and innovations in the next generation of production technology are, therefore, urgently required.

### *Dunaliella*

*D. salina* (Chlorophyta), a unicellular biflagellate green microalga, represents the most salt-tolerant eukaryotic organism. It is one of the most industrially important species of microalgae because of its extremely high β-carotene content, which accounts for up to 16% of the dry matter. The first outdoor pilot of this microalga was attempted in the USSR in 1966; however, mass cultivation on a commercial scale was first achieved in the USA and Australia in the 1980s. Currently, the large production plants are mainly established in Australia (companies, such as Western Biotechnology and Betatene) and Israel (Nature Beta Technologies). The biomass production systems mainly include open natural/artificial ponds (Fig. 5e) and raceway ponds (Fig. 5f). The mass culture of *D. salina* is mainly used for natural β-carotene production. A two-stage process has been developed in which the alga is first grown in nutrient-rich media for rapid biomass production and then transferred to nitrogen deficient media to stimulate β-carotene production. This process has been used in open raceway ponds, but is difficult to use in natural or artificial culture ponds. Today, the total annual production is estimated at 1200 metric tons of dry biomass [27].

Commercial *D. salina* is used in various forms. For example, the algal powder can be used for food and feed coloration, and β-carotene can be used for health care. Optimization of biomass production is the main bottleneck of the *D. salina* industry. In particular, existing outdoor cultivation techniques allow the density of this microalga in open ponds to reach only 8 × 10^5^ cells mL^−1^. This should be addressed by developing high-density cultivation techniques or by breeding high-yielding strains. Moreover, large-scale outdoor cultivation also means higher harvesting costs.

### *Haematococcus*

*H. pluvialis* (Chlorophyta) is a freshwater unicellular green microalga. The high content of astaxanthin (up to 4% of the dry weight) makes *H. pluvialis* attractive to biotechnologists for large-scale production in raceway ponds (Fig. 5g) or enclosed photobioreactors (Fig. 5h) at around 25–28 °C. However, the cells in open ponds systems are susceptible to contamination by other microorganisms, such as algae, fungal parasites, and zooplankton predators. Thus, a two-stage process has been developed and used for biomass production. For the first stage, green zoospores are usually cultivated in enclosed tubulars to maximize cell density. Then, the cultures are exposed to high irradiance in open ponds under nutrient stress to induce astaxanthin synthesis. Currently, *H. pluvialis* is produced in only few countries: USA (companies, such as Cyanotech), Japan (Yamaha and Biogenic, etc.), Israel (Algatech), China (Alphy and Green-A, etc.), and India (Bioprex). The total annual worldwide commercial production is estimated to be at 800 metric tons [28].

*H. pluvialis* is the main producer of natural astaxanthin. This pigment can be used as an anti-oxidant for human nutrition or as a natural colorant for the aquaculture of salmonoid fish. However, the production of astaxanthin is still restricted to that of a few hundred kilos. In fact, the *H. pluvialis* industry is still in the early stages of industrialization. This is reflected in the small number of companies capable of large-scale production and the lack of derivative products. Further expansion of biomass production will depend on the development of superior strains and a significant increase in the cells’ resistance to environmental stresses, especially fungal diseases.

### *Euglena*

*Euglena* (Euglenophyta) is the protist genus consisting of unicellular freshwater flagellates. These species can be grown photoautotrophically, heterotrophically, or photoheterotrophically, and have been studied extensively. In particular, *E. gracilis* has long been used as a model organism. This microalga has attracted the attention of cultivators as it is able to accumulate more than 50% of the dry weight as polysaccharides. The first outdoor pilot of *E. gracilis* was attempted in Japan in 2005, and the commercial cultivation was started in 2007 by Euglena Co., Ltd. (Japan) [24]. Circular ponds and raceway ponds are most common culture systems (Fig. 5i). A high cell density cultivation process, SHDP, has also been used for the mass production of biomass. Currently, only Japan and China produce *E. gracilis* commercially, with an annual production of about 70 metric tons [29, 30]. The biomass is mainly used for human nutrition. A variety of foods, drinks, and supplements containing *Euglena* have been developed as commercial products. However, the widespread cultivation and commercial application require further optimization of biomass production systems and methods.

### *Nostoc*

*N. sphaeroides* (Cyanophyta) is an edible cyanobacterium with high nutritional value. Wild *N. sphaeroides* can grow naturally both in terrestrial and aquatic environments. It has been consumed as food in China (Ge-Xian-Mi) and Peru (cushuro) for many years. The first artificial cultivation was successfully achieved in China in 2001 after some progress in breeding, and the commercial indoor production began in Changde (Hunan, China) in 2007. Currently, this microalga is cultivated only in China (Fig. 5j). The annual production is about 200 metric tons of fresh weight [6]. Many technical bottlenecks still have to be overcome to ensure further industrialization of *N. sphaeroides*. In particular, this species has high water-quality requirements and low tolerance to biological contaminants, as well as the disadvantage that fresh biomass is not easily preserved. Moreover, despite a long history of consumption, the functions and active ingredients of *N. sphaeroides* are not known, and refined techniques and products are lacking.

## Quality control standards for microalgal products

### Current status of standardization

Microalgae can convert CO_2_ into green biomass rich in lipids, sugars, proteins, carbohydrates and other valuable organic compounds. They represent one of the most promising sources of new food and functional food products, due to their balanced chemical composition. Currently, the main products commercialized or being considered for commercial applications include nutrients, polyunsaturated fatty acids, polysaccharides, phycobilins, carotenoids, vitamins, sterols, antivirals, antibiotics, and anti-cancer agents [15–18]. However, the produced microalgal biomass is subject to contamination from the entire range of heavy metals, mycotoxins, and pathogens. Contamination of products by algal toxins in mixed culture populations has also been reported. Some safety aspects of microalgae sources are intrinsic to the product, although many potential risks could also be due to production methods and conditions. The industry has largely regulated itself. The standards related to microalgae and their products are important regulations that ensure safety and quality, although urgent improvements are required. The established standards for microalgal industries are summarized in Table 6.

**Table 6:** Established standards in China, Europe and USA for microalgal industries

Species	Products	Standards in China	Standards in Europe (EU)	Standards in USA (FDA)
*Arthrospira* sp.	Powder	• Food: GB/T 16919-1997, GB/T 16919-2022 • Feed: GB/T 17243-1998	• Novel foods and novel food ingredients: No 258/97 • Larval feeding: No 440/2008 • Maximum residue levels of pesticides: No 752/2014	• Colour additive: 73.530
	Fresh* Arthrospira*	• Fresh *Arthrospira*: T/QMIS 002-2022	–	–
	Phycocyanin	• Determination of phycocyanin in *Arthrospira* powder: SN/T 1113-2002	–	–
*Chlorella* sp.	Powder	• Feed: DB32/T 565-2010, DB32/T 564-2010	• Novel foods and novel food ingredients: No 258/97 • Classification of certain goods: No 2275/88 • Feed additives: No 892/2010	–
*D. salina*	β-Carotene	• Natural carotene as food additive: GB 8821-2011 • β-Carotene as Food additive: GB 31624-2014, GB 1886.317-2021 • Determination of carotene in food: GB 5009.83-2016	• Food additives: No 231/2012	• Colour additive food: 73.95 • Drugs: 73.1095 • Cosmetics: 73.2095
*H. pluvialis*	Powder	• Food: GB/T 30893-2014	–	• Colour additives for salmonid fish feed: 73.185
	Astaxanthin	• Determination of astaxanthin in *H. pluvialis* powder: GB/T 31520-2015	• Novel foods: 2017/2470	• Colour additives for salmonid fish feed: 73.35, 73.37
*N. sphaeroides*	Dry particulates	• Food: DB42/T 1156-2016	–	–

As the world’s largest producer of microalgae, China is gradually establishing complete standards for microalgae and related products. Among them, the quality and safety standards for the use of microalgal powder as food or feed, such as those from *Spirulina* and *H. pluvialis*, have been established. Notably, the standard of fresh *Arthrospira* has been established recently. Furthermore, several standards are being developed, such as technical specifications for production, quality standards, and safety standards for each commercial microalgal species. In the EU, the approval and use of microalgae and their extracts as novel food or additives follow the New Resource Food Approval Regulation (EU) 2015/2283 and the New Resource Food Regulation (EU) 2018/102. EU has established standards for using *Arthrospira*, *Chlorella*, *H. pluvialis*, and *D. salina* as novel food or additives. Other relevant detections and quality standards are also being proposed. The standards of microalgae products in the USA are mainly in the category of colorants, such as *Arthrospira*, a colorant, *H. pluvialis*, a feed colorant, and β-carotene extracted from *D. salina*, a food colorant. In addition, the standard of β-carotene as an additive of pharmaceuticals and cosmetics has also been established in the USA. It is expected that in the next 5–10 years, a comprehensive standard system including safety, quality, and production technology specifications will be built gradually.

### Urgent improvement of the standard system

As microalgal biotechnology continues to improve and algal bioproducts become more widely used, existing standards can no longer meet the needs of the rapidly developing algal industry. Therefore, the establishment and improvement of microalgae-related standards is essential. According to the current status of standardization in the microalgae industry, the construction of the standardization system should focus on the following aspects: (i) research and development of rapid detection technologies for algal active compounds should be intensified to provide microalgae producers with simple and reliable testing methods; (ii) Timely revision of relevant industry standards to meet the development needs of new species and products of microalgae. (iii) Continuous strengthening of the function of industry associations, highlighting the importance of group standards, and stimulating the vitality of market players; (iv) International cooperation should be strengthened to promote the development of international common standards.

## Future directions and perspectives of microalgal biotechnology

- Microalgal biotechnology is limited by a few algal strains available, which indirectly reduces the diversity of commercial products. Therefore, breeding techniques should be developed to screen for high-quality algal strains that can be used for mass production.
- High cell-density culture techniques are key for reducing costs, although there is scarcity of available production facilities. Similarly, culture conditions also have to be optimized to further increase productivity.
- Quality control is an important direction for the future development of biomass production technology, in particular, the need to avoid the loss of active ingredients from microalgae under inappropriate conditions.
- The main obstacle impeding the control of biological contaminants is the lack of information regarding the biochemical pathways of contamination. The prevention of biological contaminants and development of control technologies that are both economically efficient and environmentally friendly are top priorities. The development of techniques for the detection of contaminants or toxicity factors in cultures and standards for determining the acceptable levels of toxicity in algal biomass are also necessary.
- The functional and molecular mechanisms of action of the microalgal active ingredients are not completely understood, which to some extent, affects the market positioning of microalgae and development of new applications.
- Innovative strategies are obligatory, which would help realize some potential applications of microalgae, such as genetic modification (directed evolution and rational design), to increase oil content and render microalgal biofuels commercially viable.
- The untapped bioeconomic potential of microalgae should guide the exploration of the vast undiscovered possibilities of these biotechnologically important species, such as microalgal power generation.

## Conclusions

This review systematically summarizes current biomass production technologies for commercial microalgae. We concluded that high cell-density cultivation process is important for producing biomass on commercial scale in the future, and that cost-effective processes and strategies are required for the development of microalgal harvesting. Moreover, microalgal drying should not only be cost-effective, but should also consider the quality of the product, while basic research into the control of biological contaminants should be strengthened. The article then briefly reviews the current status of commercial production of some biotechnologically important microalgae and highlights the importance of improving microalgal industry standards. In summary, it is clear that before the wider application of microalgal biomass can be achieved, significant investments in technology development and technical expertise will be required.

## Data Availability

No data were used for the research described in the article.
